# Epigenetic Profiling for Early Detection and Treatment Response Monitoring in Non–Small Cell Lung Cancer: Protocol for a Prospective Translational Biomarker Study

**DOI:** 10.2196/105338

**Published:** 2026-08-27

**Authors:** Rajiv Kumar, Safia May Farry, Mark Ezegbogu, Glen Reid, Euan James Rodger, Ben Brockway, Aniruddha Chatterjee

**Affiliations:** 1 Department of Pathology and Molecular Medicine, Faculty of Medicine - Dunedin, University of Otago, 58 Hanover Street, Hercus Building, Dunedin, 9016, New Zealand, 64 210701558; 2 Department of Medicine, Faculty of Medicine - Dunedin, University of Otago, Dunedin, New Zealand; 3 Mercy Cancer Care, Mercy Hospital, Dunedin, New Zealand; 4 Te Whatu Ora – Southern, Health New Zealand, Dunedin, New Zealand

**Keywords:** lung cancer, liquid biopsy, circulating tumor DNA, DNA methylation, epigenetics, biomarkers, transcriptomics, reduced representation bisulfite sequencing, RRBS, treatment response, Māori health, early detection

## Abstract

**Background:**

Non–small cell lung cancer (NSCLC) is the leading cause of cancer-related mortality worldwide and continues to have poor survival outcomes, with most patients diagnosed at advanced stages of disease. In New Zealand, NSCLC contributes substantially to cancer inequities, with Māori communities experiencing disproportionately high incidence and mortality rates. Although low-dose computed tomography screening can improve early detection, major limitations remain, including false-positive findings, overdiagnosis, high infrastructure costs, and limited accessibility for rural and underserved populations. Liquid biopsy approaches using circulating tumor DNA (ctDNA), particularly DNA methylation profiling, have emerged as promising, minimally invasive strategies for improving cancer detection, treatment monitoring, and precision oncology.

**Objective:**

This study aims to establish integrated genomic and epigenomic predictive and prognostic biomarkers using ctDNA, tumor tissue, and transcriptomic profiling to improve early detection, risk stratification, treatment selection and response prediction, and longitudinal monitoring, with particular emphasis on identifying molecular mechanisms associated with treatment resistance and disease progression.

**Methods:**

This prospective observational translational biomarker study is being conducted through the University of Otago and associated respiratory and oncology services in New Zealand. The study will recruit participants with NSCLC (including squamous and nonsquamous subtypes), individuals referred to fast-track lung nodule assessment clinics, and nonmalignant respiratory controls. Serial peripheral blood sampling will be performed in selected participants at predefined clinical follow-up time points to evaluate treatment response and disease progression. The availability of formalin-fixed paraffin-embedded archival tissues will be recorded, but will not be mandatory for enrollment. Genome-scale DNA methylation profiling will be performed using cell-free reduced representation bisulfite sequencing (cfRRBS), while targeted genomic profiling and transcriptomic analyses will be conducted using targeted sequencing panels and RNA sequencing. Integrative bioinformatic analyses will be used to identify molecular biomarkers associated with early-stage disease, advanced disease, treatment response, and therapeutic resistance.

**Results:**

Ethics approval for the study has been obtained from the New Zealand Health and Disability Ethics Committee (2022 EXP 12566). This study commenced in 2022, and recruitment and biospecimen collection are ongoing. The study aims to recruit approximately 450 participants, including patients with NSCLC, individuals referred through respiratory diagnostic pathways, and nonmalignant controls. As of July 31, 2026, 205 participants have been recruited, with recruitment continuing until the target sample size is reached. Molecular and data analyses are ongoing, with additional publications expected as the cohort matures.

**Conclusions:**

This study will generate one of the first integrated genomic, epigenomic, and transcriptomic liquid biopsy datasets for NSCLC in New Zealand. The findings are expected to support the development of sensitive, accessible, and equitable blood-based biomarkers for NSCLC detection and treatment monitoring while also contributing to improved precision oncology approaches and reducing NSCLC inequities among Māori populations.

## Introduction

### Background

Non–small cell lung cancer (NSCLC) is the leading cause of cancer-related mortality worldwide, accounting for approximately 1 in 5 cancer deaths. In New Zealand, more than 2000 people die from NSCLC every year. Due to its high incidence and relatively poor survival rate, it continues to have the greatest impact on overall cancer morbidity and mortality in New Zealand. Furthermore, NSCLC is associated with significant outcome inequities [1]. Therefore, a better understanding of NSCLC progression and the development of genetic and epigenetic markers for early detection and treatment guidance are important steps toward improving patient outcomes and contributing to health equity.

NSCLC is frequently diagnosed at an advanced stage, with a 5-year survival rate of approximately 15%. Low-dose computed tomography (LDCT) lung cancer screening programs represent an important opportunity to support earlier detection, improve survival outcomes, and address inequities in health care delivery [2-4]. Although LDCT has demonstrated substantial clinical value, challenges remain regarding specificity and the management of indeterminate nodules, which can lead to additional follow-up investigations [5,6]. Furthermore, distinguishing indolent disease from clinically significant disease remains difficult in some cases [7]. In addition, LDCT screening requires considerable infrastructure, specialist workforce capacity, and equitable access across geographically diverse communities [8-10]. These factors highlight the importance of developing complementary biomarkers and precision-based approaches that could enhance the effectiveness and efficiency of NSCLC screening programs.

To mitigate access, cost, and screening-accuracy issues, we propose developing a sensitive, accessible, and cost-effective blood-based test (a molecular liquid biopsy) that can detect NSCLC at an early stage, monitor patients during therapy, and predict treatment response. These molecular liquid biopsy approaches use the circulating tumor DNA (ctDNA) fraction of cell-free DNA (cfDNA) or circulating tumor cells (CTCs) isolated from plasma. Among these approaches, ctDNA has shown great promise as a dynamic molecular monitoring tool for cancer. The ctDNA test is a minimally invasive, highly specific technology that identifies the presence of a tumor from plasma-derived DNA. Its development represents a major step toward improving the diagnosis and management of cancer [11,12], with applications including earlier-stage diagnosis, detection of molecular relapses to enable more salvage therapies, monitoring treatment response, and identification of molecular targets to enable direct targeted therapies [13-19].

Tumor-derived DNA is distinguished from background normal DNA in plasma by the presence of distinct somatic DNA alterations, typically mutations and DNA methylation changes [19]. Although ctDNA analysis panels comprising common pathogenic mutations display high sensitivity for late-stage disease [20], they have only modest sensitivity for detecting early-stage disease [21]. However, recent data demonstrate that high sensitivity can be achieved using comprehensive panels that include both DNA methylation and mutation markers [22-24]. We and others have shown that alterations in DNA methylation are not only tissue- and cancer-type specific but, because of their abundance, also enable earlier tumor detection with >95% sensitivity and specificity across all stages of cancer [22-28]. In a large study (involving 123,000 samples), a tumor-specific methylation signature detected lung and other cancers at least 4 years before detection by the standard of care [29].

### Early vs Late Cancer Epigenetics

DNA methylation profiles are significantly altered in primary (early-stage) cancer compared with those of normal cells [28]. Primary tumors are globally hypomethylated and exhibit site-specific hypermethylation relative to normal cells. Late-stage tumors (metastases) generally show greater hypomethylation than primary tumors and exhibit large site-specific changes. The effect of a specific mutation on the DNA methylation profile is still a subject of research and can vary depending on tumor type or stage. We have demonstrated that it is possible to identify large and discriminatory methylation changes irrespective of driver mutation status in cancer samples [30]. Further work is needed in this area of genome biology to better understand these relationships.

### Treatment Effects on Epigenetics and the Potential for Epigenetic Analysis

In NSCLC and other oncogene-driven tumors, relapse can be driven by pre-existing (genetic) mutations or adaptive (nongenetic) resistance [31]. Adaptive resistance is a recently identified and poorly understood phenomenon in which a drug-tolerant subpopulation survives initial drug treatment. This population can eventually drive relapse via subsequent mutations or epigenetically fixed nongenetic resistance and is a recognized clinical challenge [32,33]. However, drug-tolerant cells are typically identified after prolonged drug exposure and do not represent the pre-existing population. Before drug exposure begins, it remains unknown whether cells with the tolerant phenotype are pre-existing or induced by therapy [34-36].

Our research aims to identify an epigenetic signature related to the drug-tolerant state. The drug-tolerant cells surviving initial targeted therapy consist of subpopulations of slow-cycling, stem cell–like cells. Recently, drug tolerance has been linked to distinct epigenetically controlled transcriptomic states [37]. As DNA methylation provides a somatically heritable, dynamic, and reversible epigenetic mechanism that controls gene expression in cancer cells [28], it is highly likely that DNA methylation changes contribute to the drug-tolerant state. Importantly, DNA methylation changes associated with drug tolerance have the potential to identify this subpopulation if it is present before treatment and to inform clinical management of patients with NSCLC. Furthermore, analysis of methylation changes in ctDNA provides a feasible way to monitor patients during treatment [19].

### Aims of the Study

This study has the following objectives:

1. We will establish a blood-based DNA signature (combining methylation and mutation markers) to identify NSCLC with high sensitivity and specificity. To achieve this, we will carry out sequencing-based analysis of critical gene mutations and DNA methylation landscapes in matched blood and tissue samples from patients with NSCLC, blood samples from patients referred to fast-track nodule follow-up clinics or chest X-ray clinics, and blood samples from healthy or nonmalignant controls.
2. We aim to establish a panel of epigenetic and genetic analyses to advance the prediction of treatment response in patients with NSCLC. For this, we aim to identify differences in DNA methylation, gene expression, and targeted mutations between responders and nonresponders with NSCLC receiving targeted therapy or immunotherapy.

## Methods

### Study Design and Setting

**Figure 1.**
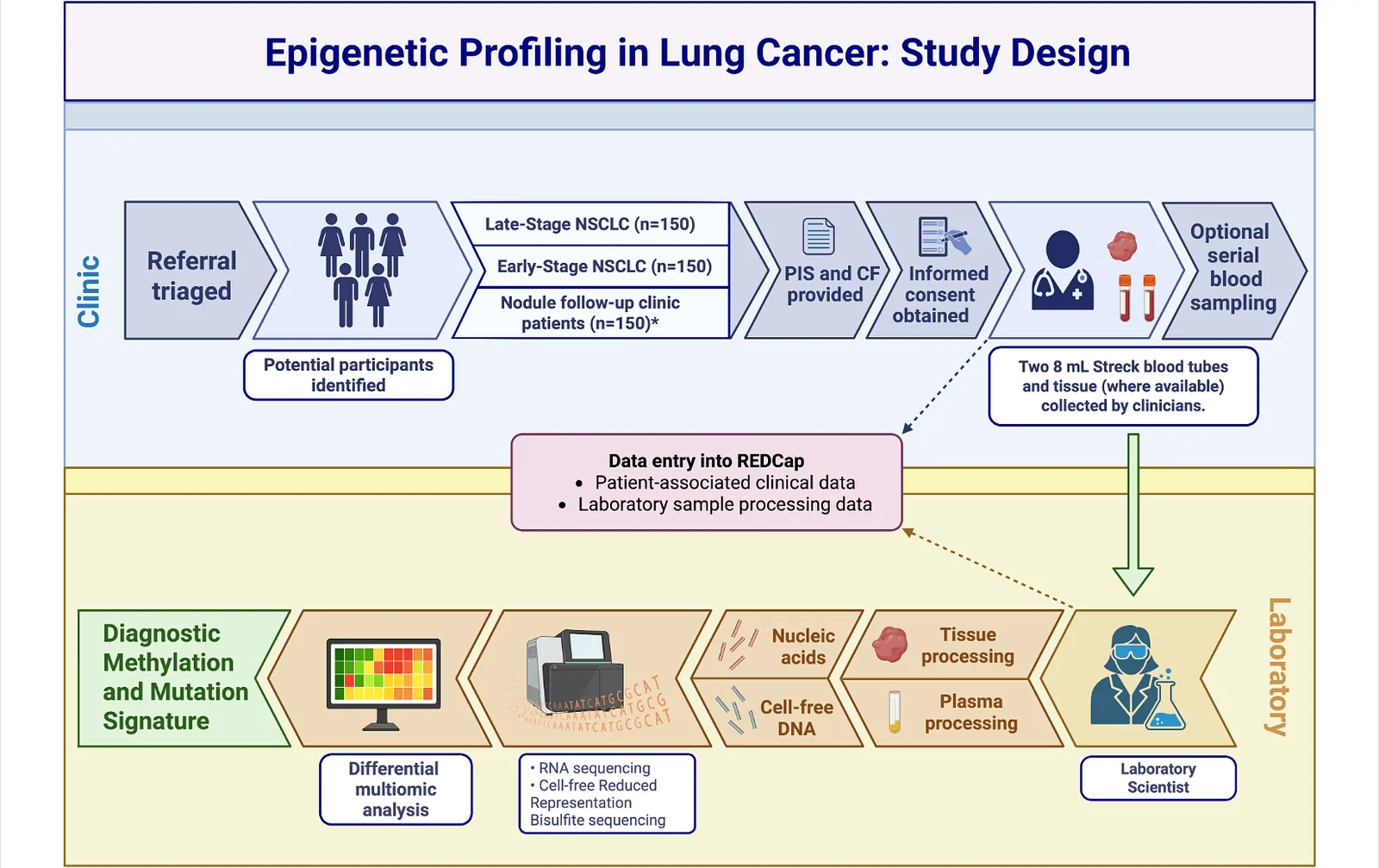
Schematic overview of patient sample collection, processing, and analysis. Patient referrals are triaged and assessed for suitability for participation. Potentially eligible participants are provided with a patient information sheet (PIS) and consent form (CF), followed by the provision of informed consent if they agree to participate. Two 8 mL Streck blood tubes and tissue (where available) are collected from eligible participants by collaborating clinicians from multiple localities, where suitability for serial blood sampling is assessed for future follow-up visits. Patient samples are processed by laboratory scientists for the extraction of nucleic acids. Collaborating clinicians enter patient-associated clinical data into REDCap, and laboratory scientists enter sample processing details into REDCap. Extracted nucleic acids are then further processed in preparation for sequencing. Following sequencing, the data are analyzed and integrated with the corresponding clinical information for each patient to provide multimodal insights. Participants include individuals referred to nodule follow-up clinics and self-declared nonmalignant controls.

This prospective observational translational biomarker study investigates genomic and epigenomic biomarkers in patients with NSCLC and control participants. The study integrates longitudinal liquid biopsy sampling, tissue-based molecular analyses, and clinical data collection (Figure 1). The study is sponsored through the Department of Pathology and Molecular Medicine and the Department of Medicine, Faculty of Medicine (Dunedin), at the University of Otago, in collaboration with Dunedin Hospital and associated respiratory clinics, including fast-track lung nodule assessment pathways. In addition, we have expanded the locality approval to include the Cancer and Chronic Conditions Research Unit in the Waikato District (North Island of New Zealand) for sample collection.

### Study Population

The study will include nonmalignant controls and patients with precancerous symptoms or early- and late-stage NSCLC (treated or untreated). The complete eligibility criteria are reported in Textbox 1.

Textbox 1. **Eligibility criteria for participation in the study.**

**Inclusion criteria**

- Aged ≥18 years

- Histological or cytological diagnosis of non–small cell lung cancer, including nonsquamous or squamous cell carcinoma

- One of the following participant groups:

- Early- or advanced-stage (stage IIIB or IV) disease, according to the American Joint Committee on Cancer Staging Manual, eighth edition
- Patients referred to nodule follow-up clinics or chest X-ray clinics
- Nonmalignant control participants

**Exclusion criteria**

- Unable to provide informed consent

### Recruitment

Potential participants are identified through respiratory services, fast-track lung cancer assessment clinics, chest X-ray clinics, oncology services, and respiratory research databases. Recruitment occurs in both Dunedin and Invercargill. Patients referred for respiratory assessment undergo routine clinical evaluation, including imaging and diagnostic procedures as clinically indicated. Participants are approached regarding study participation during routine clinical care.

### Ethical Considerations

Patients identified as meeting the inclusion criteria will be approached for recruitment into the study and asked to provide informed consent. Informed consent will be obtained for the use of their archival tissue samples and blood samples for genomic and epigenomic analyses. This study will be conducted in full conformance with the principles of the Declaration of Helsinki, Good Clinical Practice (GCP), the Code of Rights of the Health and Disability Commissioner, and the laws and regulations of New Zealand.

All research involving human biological samples is conducted in a manner that respects indigenous values, including the preservation of *mana* (prestige or spiritual power) and adherence to *tikanga* (cultural practices). Both biological samples and the data associated with each participant are handled within a culturally competent framework, recognizing that biological samples are *taonga* (treasured) and that data generated from these samples are *tapu* (sacred or restricted) and must be handled accordingly. *Tikanga* is upheld throughout the study, including culturally appropriate sample storage and disposal procedures. Where requested by participants through the consent process, a *karakia* (blessing) may also be performed during sample disposal.

Ethics approval for the study has been obtained from the New Zealand Health and Disability Ethics Committee (2022 EXP 12566).

### Biospecimen Collection and Sample Size

Blood samples are collected from participants at baseline and at longitudinal follow-up time points during clinical management, as applicable. Peripheral blood samples are collected in 8 mL Streck tubes. Serial blood sampling may occur approximately every 6 to 12 months for up to 3 years during routine clinical follow-up, as feasible. Optional archival formalin-fixed, paraffin-embedded (FFPE) tissue samples or fresh-frozen tumor tissue samples are obtained from diagnostic procedures. Tumor-rich regions are identified by pathological review. The study aims to recruit approximately 450 participants, including (1) patients with early-stage NSCLC, (2) patients with advanced-stage NSCLC, (3) participants referred to nodule follow-up clinics, and (4) nonmalignant controls. Nonmalignant controls will comprise self-declared healthy individuals with no known medical comorbidities. These participants will be recruited opportunistically rather than through targeted matching. For all participant groups, relevant patient, tumor, and treatment characteristics will be recorded where available and considered in downstream analyses, where appropriate, for this discovery cohort study.

### Plasma and Tissue Sample Processing

Plasma is isolated within 1 week of blood collection to minimize cellular lysis. Blood samples are centrifuged, plasma is retained and stored at −80 °C, and buffy coat fractions are preserved for matched nucleic acid analyses. cfDNA is extracted using standardized protocols and subjected to downstream genomic and epigenomic analyses [38-40]. Tumor tissues undergo macrodissection, where required, to enrich tumor cellularity. DNA and RNA are isolated using commercially available extraction kits. Quality control and nucleic acid quantification are performed using fluorometric and electrophoretic methods.

### DNA Methylation Analysis

We will use cell-free reduced representation bisulfite sequencing (cfRRBS) to obtain genome-scale DNA methylomes and identify genomic regions with the largest methylation differences between the 2 groups. Our team has extensive experience and is internationally recognized for analyzing epigenomic data [30,41-48]. We will use our well-established laboratory and computational methods [43-46,49-51] to perform library preparation, data processing, and statistical testing to identify differential methylation. We will compare ctDNA methylomes from nonmalignant controls and patients with NSCLC using our established analysis pipeline [45,46]. We will use ANOVA to determine statistical significance and subsequently apply multiple-testing correction, as appropriate, to detect differentially methylated regions (DMRs) associated with tumors. In addition, we will compare our reduced representation bisulfite sequencing (RRBS) methylomes with The Cancer Genome Atlas (TCGA; array-based methylation data from 585 primary lung tumors are available) and independent ctDNA RRBS datasets from 29 patients with NSCLC and 75 healthy controls (13 million reads per sample; GEO accession GSE79279) [22].

At 100X coverage, the predicted sensitivity for detecting 10 driver methylation changes is 99% with a 1% abundance of ctDNA, and at 100X coverage, the sensitivity for detecting 100 driver methylation changes is 99% with a 0.1% abundance of ctDNA [25]. Furthermore, the observed and expected numbers of DMRs and the detected signals showed a near-perfect linear association when ctDNA was diluted to 0.001% of total cfDNA (*r*^2^=0.99) [25]. We will compare ctDNA methylomes from participants without malignancy and patients with NSCLC using our established analysis pipeline [45,46] to detect DMRs that indicate the presence of a tumor. The inclusion of participants without malignancy will allow the identification of methylation changes specific to lung tumors rather than to other respiratory illnesses. We will not sequence germ-line DNA in this work. Our primary aim is to identify large-scale DNA methylation changes specific to NSCLC.

### Mutation Profiling

After DNA methylation profiling, when additional DNA remains from a sample, we aim to perform mutation profiling on a subset of samples. Targeted mutation profiling will be performed using clinically relevant next-generation sequencing (NGS) panels designed to detect key genomic alterations in NSCLC, including actionable driver mutations. These assays will enable comprehensive characterization of molecular features associated with NSCLC detection, tumor biology, treatment response, and mechanisms of therapeutic resistance [52,53]. Particular focus will be placed on clinically relevant alterations in genes commonly implicated in NSCLC pathogenesis and targeted therapy response, including epidermal growth factor receptor (EGFR), Kirsten rat sarcoma (KRAS), V-Raf murine sarcoma viral oncogene homolog B (BRAF), anaplastic lymphoma kinase (ALK), c-ros oncogene 1 (ROS1), mesenchymal epithelial transition (MET)*,* and tumor protein p53 (TP53). Mutation profiling will also be integrated with epigenetic analyses to explore combined genomic and methylomic signatures associated with disease progression, immunotherapy response, and resistance to targeted therapies. Where longitudinal samples are available, temporal changes in mutation profiles will be evaluated to investigate tumor evolution and treatment-associated molecular adaptations.

### Transcriptomic Analysis

RNA sequencing (RNA-Seq) data will be generated using our well-established pipelines for clinical tissue samples (where tissue samples are available), including those with low RNA input [30,44,54-58]. Briefly, TruSeq (Illumina Inc) total RNA-Seq libraries will be prepared, yielding 60 to 80 million paired-end reads per sample. Sequence reads will be processed and aligned to the reference genome as previously described [58], and gene expression will be quantified using the transcripts per million (TPM) method after correcting for multiple mapping and fragment bias [59]. Differential expression will be determined with *DESeq2* [39], with *P* values adjusted for multiple testing using the Benjamini-Hochberg method (false discovery rate [FDR] <0.05). Transcriptomic profiles will be analyzed using the gene set enrichment analysis (GSEA) against specific molecular signatures (MSigDB) and integrated with the methylome data, as we have done previously to identify epigenetic drivers associated with transcriptional changes [28,42,56].

### RRBS and RNA-Seq Analysis of FFPE Tissues

From pretreatment tumor samples, tumor DNA and RNA will be used to perform RRBS and RNA-Seq. We have developed these methods for both cell lines, tissues, and small amounts of FFPE material. We have demonstrated high reproducibility of RRBS methylation data from FFPE tissues [48,60] and have also validated methylomes using independent methods [42]. In addition, we have demonstrated robust expression profiling using RNA isolated from small amounts of FFPE tissue in multiple tumor types [58]. Our expertise in the regular use of these methods will enable us to successfully profile lung tumors and identify markers of therapy resistance.

### Performance Assessment

A central challenge in this study is the selection and validation of candidate markers. In the discovery phase, candidate markers will be chosen from DMRs reaching statistical significance (FDR<0.05) and demonstrating a methylation difference between groups of >20%.

Clinical performance will then be assessed by testing the correlation with treatment response, using receiver operating characteristic (ROC) curve analysis. As a benchmark, for our second aim, the programmed death-ligand 1 (PD-L1) tumor proportion score has variable and modest predictive value, with an area under the curve (AUC) of approximately 0.68 for predicting immunotherapy response [61]. An AUC exceeding this threshold would therefore be considered clinically meaningful in our cohorts, indicating superior predictive performance relative to PD-L1 alone, and would provide the basis for future translational work. Biomarkers meeting this criterion will proceed to validation in independent external cohorts before prospective evaluation within a clinical trial to establish their utility in guiding treatment selection.

### Clinical Data Collection and Management

Clinical and pathological information collected includes the following:

- Demographic variables
- Ethnicity
- Histological subtype
- Disease stage according to the American Joint Committee on Cancer (AJCC)–Union for International Cancer Control (UICC) TNM Staging System (eighth or ninth edition, as applicable)
- Treatment information
- Treatment response
- Longitudinal clinical outcomes

Clinical data are linked to molecular data using deidentified participant identifiers and are collected and managed in REDCap (Vanderbilt University), provided by the University of Otago for staff and students. REDCap is a secure, web-based application designed to support data capture for research studies and provides role-based permissions, secure institutional hosting, audit logging, and controlled data export functionality [62]. The REDCap database, as designed for the Lung Epigenetics Study, is overseen by designated project administrators who are responsible for maintaining database integrity, managing user permissions, and ensuring compliance with institutional data governance policies.

**Figure 2.**
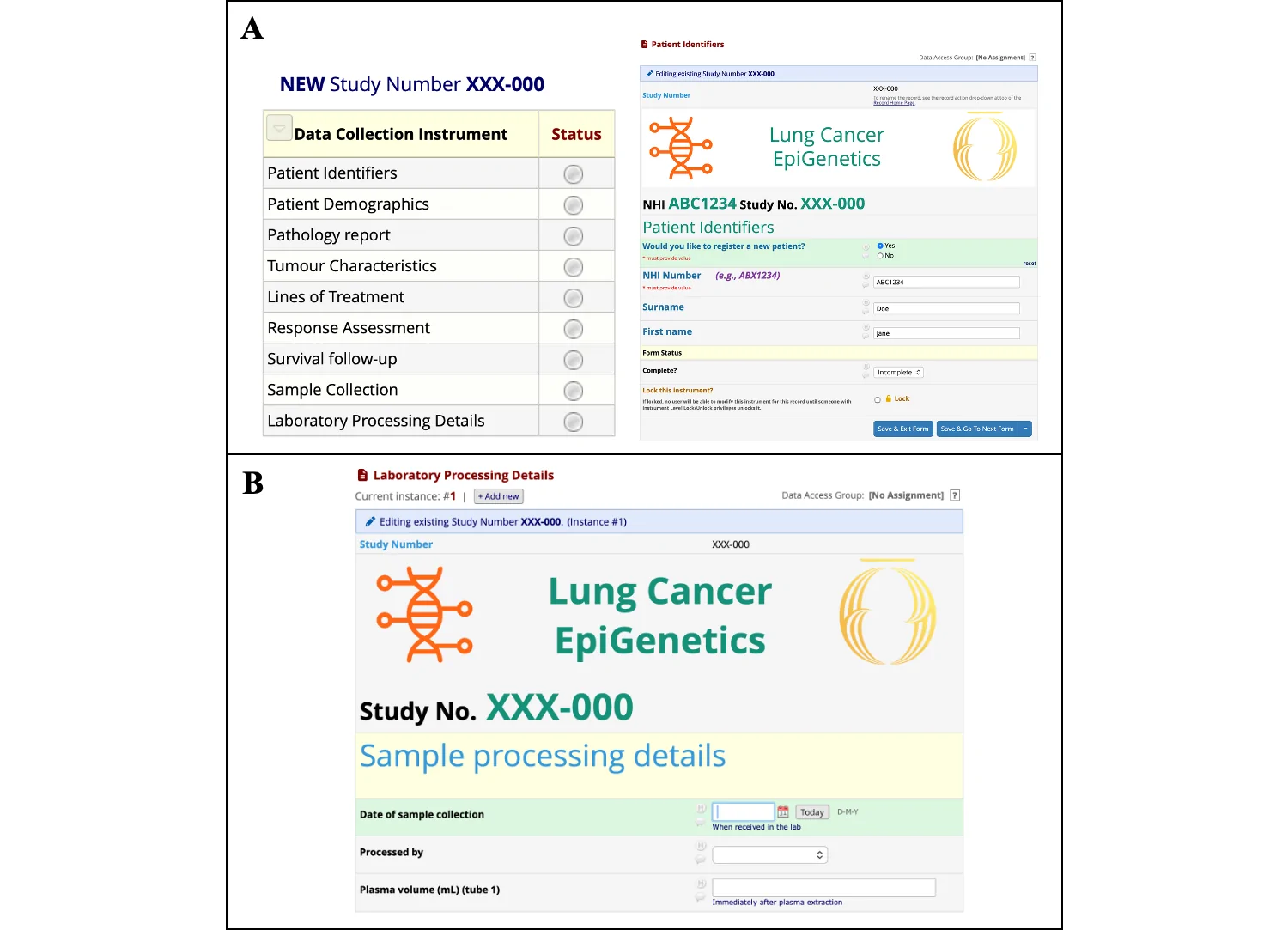
Example screenshots of clinical data input of a new patient in the Lung Cancer Epigenetics REDCap database. (A) Summary of the data collection instruments and overview of the collection fields in the patient identifiers form to be completed by collaborating clinicians (data entry user role). (B) Overview of the laboratory processing details form to be completed by laboratory scientists (laboratory scientist user role).

Access to the REDCap database is controlled using predefined user roles, which determine the actions that users are permitted to perform within the database and help ensure that users have access only to the data required for their responsibilities within the project. Users assigned to the admin-level role have full administrative access to the database. Data entry roles are assigned to clinical collaborators responsible for entering patient and clinical data (Figure 2A) from specific contributing sites and updating records as new clinical information becomes available. Users in these roles are restricted to their assigned data access group (DAG), meaning that they cannot view or edit records from other participating localities. Users assigned to the laboratory scientist role are responsible for entering and managing laboratory-related research data. This includes editing sample collection and laboratory processing details (Figure 2B) associated with each deidentified participant identifier. Laboratory scientists do not have permission to view real patient identifiers (National Health Index [NHI] number, name, or date of birth). The auditor role provides read-only access to the database for users who require oversight of the database, excluding patient identifiers, without the ability to modify records.

An additional functionality of REDCap is the ability for users with the admin-level role to “lock” records, meaning that records can no longer be modified. This ensures data integrity by preventing further edits once records have been finalized, thereby reducing the risk of accidental or unauthorized changes and maintaining data accuracy for downstream analysis and reporting. To further ensure accuracy, exports of associated clinical data are conducted on a quarterly basis so that downstream analyses are consistently linked to a defined, time-stamped version of the dataset, accounting for the possibility that clinical data may be updated over time.

## Results

Participant recruitment and biospecimen collection are ongoing. The study commenced in 2022 following funding, with recruitment and data collection continuing until the target sample size is reached. As of July 31, 2026, 205 participants have been recruited. Molecular analyses, including DNA methylation profiling, targeted sequencing, and transcriptomic analyses, have commenced, with data analysis progressing alongside recruitment. The first publication describing the technical methodology and feasibility of the study has been published, and analyses addressing the study objectives are ongoing as the dataset matures. Additional findings will be reported through future publications, with further outputs anticipated from 2027 onwards. The study is expected to generate one of the first integrated NSCLC methylome datasets in New Zealand, including valuable liquid biopsy profiling. Continued nationwide recruitment will maximize the value of this collaborative lung cancer resource by enabling robust analyses across multiple research objectives and supporting future translational research.

To support these analyses, it was also necessary to develop low-input analysis methods and perform an assessment to choose an appropriate omics platform for genome-scale DNA methylation analysis. Our comparative analysis demonstrated that both enzymatic and bisulfite-based approaches have distinct advantages for cfDNA methylation profiling in liquid biopsy applications [63]. Enzymatic methyl sequencing (EM-Seq) provided higher mapping efficiency, broader genomic coverage, and improved CpG detection at lower coverage thresholds, whereas bisulfite-based methods showed higher conversion efficiency, superior reproducibility, lower cost, and better coverage of biologically relevant regions such as promoters and exons. Overall, cfRRBS emerged as the most balanced approach in terms of cost-effectiveness, accuracy, and reproducibility, supporting its strong potential for translational cfDNA methylation studies and clinical liquid biopsy applications. Therefore, we will use cfRRBS to analyze NSCLC samples in this study.

## Discussion

### Blood-Based Biomarkers for Improved NSCLC Outcomes

Against the backdrop of the disproportionately high burden of NSCLC in New Zealand, improving the accuracy of tumor detection and individual risk stratification could inform decisions about personalized investigation, treatment, and monitoring strategies. This could result in improved clinical management and enhanced outcomes for patients with NSCLC. To mitigate the issues of access, cost, and accuracy of screening, the development of blood-based DNA methylation, DNA mutation, and gene expression–based assays that are sensitive, accessible, and cost-effective could improve the detection of lung tumors, assist in treatment management, and contribute to reducing the health care burden and improving NSCLC outcomes.

### Molecular Risk Stratification to Support Future Lung Cancer Screening

Although LDCT remains an important and clinically valuable approach for lung cancer screening, integrating complementary molecular biomarkers may further enhance screening efficiency, accessibility, and precision. A New Zealand–based analysis estimated the cost-effectiveness of biennial national LDCT screening at approximately NZ $145,000 (NZ $1=US $0.59, as of July 31, 2026) per quality-adjusted life-year gained [10], highlighting the importance of developing additional strategies that optimize screening pathways and patient selection within resource-constrained health systems. Current international LDCT eligibility criteria typically focus on individuals aged 55 to 75 years with a substantial smoking history [7]. However, NSCLC is increasingly being identified in younger individuals and in never smokers [64,65], populations that may fall outside existing screening frameworks. In this context, ctDNA-based methylation assays could serve as a complementary tool to support risk stratification and prioritization of individuals, particularly those who may not meet conventional criteria but remain at elevated risk. Importantly, ctDNA-based testing has the potential to be relatively cost-effective and scalable as technologies continue to mature.

### Epigenetics as an Accessible Tool for Precision NSCLC Investigation

A molecular epigenetic test that can distinguish between nonmalignant disease, nonspecific computed tomography (CT) findings, and clinically significant disease would reduce the number of people requiring follow-up, leading to reduced anxiety and lower costs to the health care system. As ctDNA analysis only requires a 10 mL peripheral blood sample, it enables sampling to be carried out within the community by rural nurses, marae-based clinics, and general practices at any time, greatly improving accessibility and reducing geographic and ethnic inequities.

### Epigenetic and Molecular Biomarkers for NSCLC Progression and Therapeutic Resistance

In addition to its use as an adjunct diagnostic tool, a customized ctDNA panel could be used to monitor treatment response (by providing molecular evidence of relapsed disease [18]) and guide treatment decisions. Identification of these markers at the time of diagnosis would be cost-effective and provide the necessary foundation for patients with NSCLC in New Zealand to receive care consistent with international best practice. The objective response rate for targeted therapy in NSCLC varies from 50% to 70% [66-69]. However, most patients develop progressive disease after 18 months of treatment. The development of acquired resistance not only shortens survival but also limits the long-term efficacy of targeted therapies. For patients with NSCLC harboring EGFR mutations who develop resistance to targeted therapy, a second-line therapy is often not available. In New Zealand, osimertinib is funded as the first-line treatment for patients with advanced NSCLC harboring EGFR mutations [68]. Similarly, for patients whose tumors harbor ALK mutations, there is no targeted second-line therapy after they develop resistance to alectinib. The development of markers of tolerance to targeted therapies could enable better clinical management of patients with NSCLC, contributing significantly to reducing the health burden in New Zealand, particularly for patients who lack access to specialized health care, thereby addressing the disparities in outcomes. Furthermore, the results of this project might also provide a basis and opportunity to treat patients with NSCLC with new, lower-toxicity epigenetic drugs (such as guadecitabine, currently being evaluated in 4 active clinical trials for NSCLC) to prevent future tumor progression. Integrative analysis of DNA methylome, expression patterns, and mutation patterns will provide information on the drivers of NSCLC progression and provide a basis for future studies. Using these profiles, epigenetic drivers and pathways that play a role in tumor progression and metastasis in NSCLC can be identified. A plethora of tools is available in the laboratory to study these experimentally and demonstrate how they contribute to cancer progression and metastasis.

As an observational biomarker study, findings from this discovery cohort will require external validation in larger independent cohorts. This study is designed to generate important preliminary insights and identify promising epigenetic biomarkers in NSCLC. Substantially larger independent validation cohorts will enable us to assess the robustness, reproducibility, and generalizability of the identified markers across diverse patient populations and clinical settings [70]. Larger biomarker-driven clinical studies will also be necessary to more confidently evaluate associations with translational outcomes and measures of efficacy, such as treatment response and survival.

Another limitation concerns the paired analysis of tissue- and blood-derived analytes from the same patients. In NSCLC, diagnostic biopsies are often small and clinically prioritized for routine histopathological and molecular testing, leaving limited residual material available for research purposes. Consequently, matched tissue and blood samples will not always be comprehensively analyzed for all participants, which may reduce the ability to directly correlate tissue-derived and ctDNA epigenomic profiles.

Furthermore, variability in cfDNA yield between patient plasma samples represents a technical challenge. It is well established that cfDNA concentration can vary substantially depending on tumor burden, disease stage, biological variability, and preanalytical factors [71]. Samples with very low cfDNA input may not provide sufficient material for robust genomic or epigenomic profiling, potentially affecting assay performance, data completeness, and downstream analyses. However, we have now developed sensitive, low-input methods for methylation analysis to address this challenge [63].

### Conclusions

Strengths of this study include prospective longitudinal sampling; the integration of genomic, epigenomic, and transcriptomic analyses; the inclusion of participants across the diagnostic and treatment pathway; and the incorporation of equity-focused approaches relevant to Māori health outcomes in New Zealand. This prospective translational study will provide important insights into the genomic and epigenomic landscape of NSCLC in New Zealand. By integrating ctDNA methylation profiling, mutation analysis, and transcriptomic data, the study seeks to establish clinically relevant biomarkers for early detection and treatment monitoring while supporting equitable precision oncology approaches.

## Abbreviations

AJCC: American Joint Committee on Cancer
ALK: anaplastic lymphoma kinase
AUC: area under the curve
BRAF: V-Raf murine sarcoma viral oncogene homolog B
cfDNA: cell-free DNA
cfRRBS: cell-free reduced representation bisulfite sequencing
CT: computed tomography
CTC: circulating tumor cell
ctDNA: circulating tumor DNA
DAG: data access group
DMR: differentially methylated region
EGFR: epidermal growth factor receptor
EM-Seq: enzymatic methyl sequencing
FDR: false discovery rate
FFPE: formalin-fixed, paraffin-embedded
GCP: Good Clinical Practice
GSEA: gene set enrichment analysis
KRAS: Kirsten rat sarcoma
LDCT: low-dose computed tomography
MET: mesenchymal epithelial transition
NGS: next-generation sequencing
NHI: National Health Index
NSCLC: non–small cell lung cancer
PD-L1: programmed death-ligand 1
RNA-Seq: RNA sequencing
ROC: receiver operating characteristic
ROS1: c-ros oncogene 1
RRBS: reduced representation bisulfite sequencing
TCGA: The Cancer Genome Atlas
TP53: tumor protein p53
TPM: transcripts per million
UICC: Union for International Cancer Control
